# Comprehensive Evaluation of 24 Red Raspberry Varieties in Northeast China Based on Nutrition and Taste

**DOI:** 10.3390/foods11203232

**Published:** 2022-10-16

**Authors:** Yiping Yu, Guang Yang, Lanying Sun, Xingshun Song, Yihong Bao, Ting Luo, Jinling Wang

**Affiliations:** 1 School of Forestry, Northeast Forestry University, No. 26, Hexing St., Harbin 150040, China; 2 Institute of Rural Revitalization Science and Technology, Heilongjiang Academy of Agricultural Science, No. 800, Chuangxinsan St., Harbin 150027, China; 3 School of Life Sciences, Northeast Forestry University, No. 26, Hexing St., Harbin 150040, China; 4 State Key Laboratory of Food Science and Technology, Nanchang University, No. 999, Xuefu St., Nanchang 330047, China; 5 Key Laboratory of Forest Food Resources Utilization of Heilongjiang Province, No. 26, Hexing St., Harbin 150040, China

**Keywords:** red raspberry, physicochemical properties, sensory characteristics, principal component analysis, cluster analysis

## Abstract

Red raspberry is a kind of fruit with high nutritional values. To evaluate the comprehensive quality of 24 red raspberry varieties in Northeast China, physicochemical properties, bioactive compounds and sensory characteristics were measured, followed by principal component analysis (PCA) and cluster analysis (CA). Altogether, eight important property indexes for processing attributes were selected out using PCA, including titratable acidity (TAC), sugar-acid ratio (SAR), pH, length, diameter, weight, sucrose and citric acid. Six individual sugars, including l-rhamnose monohydrate, fructose, glucose, sucrose, maltose and d-trehalose anhydrous, as well as eight organic acids, including oxalic acid, tartaric acid, malic acid, α-ketoglutaric acid, lactic acid, citric acid, fumaric acid and succinic acid, were identified in red raspberry. The two main clusters according to individual sugar, organic acids and SAR indicated that varieties including ‘European red’, ‘DNS9’, ‘Bulgaskc’, ‘Canby’ and ‘Samodiva’ were suitable for fresh-eating or processing to juice or other products directly because they had suitable SAR; other varieties with relatively low SAR were unsuitable for fresh-eating and need to adjust their excessive sour taste during processing.

## 1. Introduction

The red raspberry (*Rubus idaeus* L.) belongs to the Rosaceae family and is widely cultivated in Asia [1]. It is known to have excellent cold resistance, disease resistance, nutritional values and flavor, in addition to widespread market availability [2]. Extensive studies are actively carried out to investigate the components and efficacy of red raspberry. These small and soft fruits have high levels of nutrients, especially individual sugar, organic acids, vitamins, and phytochemical compounds [3]. Phenolic compounds such as phenolic acids [4], flavonoids such as anthocyanins and flavonols [5], and tannins [6] are responsible for various health benefits associated with humans, including prevention of inflammation [7], being against colonic epithelial damage [8], as well as being anti-diabetic [9,10], anti-cardiovascular diseases and anti-cancer [11]. Red raspberry can be consumed fresh, however, one of the main constraints for consuming and marketing red raspberry fruits is the short shelf-life, due to the high perishability of the fruits. Long shelf-life processed products, such as jams, purees, juices, marmalades, and jellies are the main products of raspberry fruits [12].

It is particularly important to evaluate the quality of red raspberry, which mainly includes physical, chemical characteristics and sensory characteristics, for the products development and breeding programs. There were studies [13] that have evaluated the physical and chemical properties of the fruits as titratable acidity (TAC), soluble solids contents (SSC), sugar-acid ratio (SAR) and shelf life of 7 red raspberry cultivars. The results showed that TAC, SSC and SAR of 7 red raspberry varieties ranged from 1.49 to 2.08%, 8.30 to 12.20 °Brix and 4.50 to 5.70, respectively. The varieties ‘Imara’ and ‘Kweli’ were known to give a high yield potential and excellent berry quality, since they had a good growth habit, large fruit, and excellent berry quality and shelf-life. There have been studies which [14] determined the physicochemical properties, such as color, TAC, SSC, reducing sugar, total sugar and SAR, in five growth stages of three commercial raspberries. The results showed that ripe raspberries (stages 3 and 4) with a high anthocyanin level and SAR were suitable for fresh use and processing. Chen et al. [15] reported the physicochemical properties such as color, weight, length, diameter, TAC, SSC, ascorbic acid and total sugar of 17 genotypes of red raspberry and drew the conclusion that the higher consumer acceptance related with higher ratio of SAR. There have been studies [16] which assessed the physicochemical properties including individual sugar and organic acids of 14 wild red raspberry varieties. The results showed that individual sugar distribution of red raspberry was dominated by fructose, glucose and sucrose (mean value of 32.20, 24.30 and 9.10 g/kg fresh weight (FW)), and citric acid was the main organic acids in red raspberry (mean value of 13.10 g/kg FW). There were large differences found among the varieties such as the ‘A2’, ‘A9’, ‘A12’ and ‘A14’ varieties, that might be used in future breeding programs.

The aim of this study was to assess the physicochemical, bioactive compounds and sensory characteristics of 24 red raspberry varieties from Northeast China. In addition, principal component analysis (PCA) and cluster analysis (CA) were used to access their comprehensive quality. Results of this study could not only provide references for geneticists to develop varieties with higher nutritional value and consumer acceptability, but also help growers choose the best germplasm resources for fresh-eating and processing fruits with improved quality.

## 2. Materials and Methods

### 2.1. Chemicals

All the standards for high performance liquid chromatography (HPLC), including l-rhamnose monohydrate, fructose, glucose, sucrose, maltose, d-trehalose anhydrous, oxalic acid, tartaric acid, malic acid, α-ketoglutaric acid, lactic acid, citric acid, fumaric acid, succinic acid, ascorbic acid (HPLC grade) were obtained from Shanghai YuanYe Biotechnology (Shanghai, China). Other chemicals were obtained from Tianjin Guangfu Chemical Reagent (Tianjin, China). All reagents were either analytical grade or HPLC grade.

### 2.2. Sample Preparation

The red raspberry was obtained from Heilongjiang Academy of Agricultural Sciences (Harbin, China) in July 2021. The 24 red raspberry varieties were introduced from various regions and countries, planted and cultivated in Heilongjiang Academy of Agricultural Sciences. Detailed information of the samples was presented in Table 1. All of the fruits were hand-picked at commercial maturity. Some of the harvested fruits were immediately used for the determination of fruit length, diameter, weight, moisture. The other fruits were kept in icebox and transported to the laboratory within 2 h, flash-frozen and stored at −20 °C until use.

Red raspberry fruits (500 g) were squeezed after natural thawing, filtered using an 8-layer gauze, then the gauze was washed twice with distilled water. The washing solution and red raspberry juice were mixed and the mixture was brought to 500 mL using distilled water. The mixture (15 mL) was centrifuged at 4000 r/min, 4 °C for 15 min (TGL-16C, Sand Eagle Scientific Instruments Co. Ltd.; Shanghai, China), then filtered through a 0.45 μm membrane to obtain a supernatant for further determination of individual sugar, organic acids, ascorbic acid, et al.

### 2.3. Determination of Fruit Length, Diameter, Weight

The length and diameter of red raspberry fruits (*n* = 50) were measured by a vernier caliper. The weight of red raspberry fruits (*n* = 50) was measured by an electronic balance (JA2003N, Shanghai Yoke Instrument System Co. Ltd.; Shanghai, China) [17].

### 2.4. Determination of Titratable Acidity (TAC), Soluble Solids Contents (SSC), pH and Sugar-Acid Ratio (SAR)

Moisture, titratable acidity (TAC), soluble solids contents (SSC) and pH values were analyzed following the method of the association of official analytical chemists (AOAC) [18]. Total sugar contents were analyzed following the method of the phenol-sulfuric acid method [19]. Reducing sugar contents were analyzed following the method of the 3,5-dinitrosalicylic acid method [14]. The sugar-acid ratio (SAR) was the ratio of SSC and TAC.

### 2.5. Determination of Individual Sugar

Individual sugar, including l-rhamnose monohydrate, fructose, glucose, sucrose, maltose and d-trehalose anhydrous, was measured by the HPLC method according to Tilahun et al. [20], with slight modifications. A total of 1 mL of supernatant was transferred to HPLC vials and analyzed. The system was carried out on a Waters HPLC (Waters Technologies Inc., Milford, CT, USA) equipped with a 2414 RID detector and a 5NH_2_-MS column (4.6 × 250 mm, 5 µm). The mobile phase was consisted of acetonitrile and deionized water (75:25, *v*/*v*). The flow rate for mobile phase was 1.0 mL/min. The injection volume was 20 μL. The column oven temperature was maintained at 37 °C. The elution time was 30 min. Identification of the sugar was carried out by comparison of their retention time with those of the standards. Standard curves were obtained using 0–20 mg /mL sugar standards. Individual sugar contents in samples were calculated by regression equation and expressed as g/100 g dry weight (DW).

### 2.6. Determination of Organic Acids

Organic acids, including oxalic acid, tartaric acid, malic acid, α-ketoglutaric acid, lactic acid, citric acid, fumaric acid and succinic acid, were measured by the HPLC method according to He et al. [21]. The system was carried out on a Agilent 1260 Infinity II HPLC equipped with a DAD detector (Agilent Technologies Inc., Santa Clara, CA, USA) and a Agilent Poroshell 120 EC-C18 column (4.6 × 150 mm, 4 µm). Two eluents, filtered through a 0.45 µm durapore membrane, were used as mobile phases: KH_2_PO_4_ (0.5%, *w*/*v*, pH 2.3, eluent A), methanol (eluent B). The elution condition was isostatic elution with A:B of 97:3 (*v*/*v*). The flow rate for mobile phase was 0.7 mL/min, and the injection volume was 10 μL. The column oven temperature was maintained at 35 °C. Wavelength for the detection was 210 nm. Organic acid contents in samples were calculated by regression equation and expressed as g/100 g DW.

### 2.7. Sensory Characteristics Analysis

Sensory characteristics evaluation of red raspberry was conducted using a 9-point hedonic scale [22], with slight modifications. Sensory characteristics evaluation was performed in Food Nutrition laboratory, Department of Food Science and Engineering. In total, 24 red raspberry varieties were sensory evaluated by 100 trained judges (50 males and 50 females, from 17 to 65 years old) selected from students and teaching staff of the Northeast Forestry University. The participants were asked to evaluate each sample for sensory attribute’s appearance, color, flavor, taste, and overall acceptability. Each attribute was scored by following 9-point scale as follows: 9 = Like extremely; 8 = Like very much; 7 = Like; 6 = Like slightly; 5 = Neither like nor dislike; 4 = Dislike slightly; 3 = Dislike moderately; 2 = Dislike; 1 = Dislike extremely.

### 2.8. Determination of Ascorbic Acid

Ascorbic acid was measured by the HPLC method according to Pantelidis et al. [23], with slight modifications. The system was carried out on a Agilent 1260 Infinity II HPLC equipped with a DAD detector (Agilent Technologies Inc., Santa Clara, CA, USA) with a Agilent Extend-C18 column (4.6 × 250 mm, 5 µm). The mobile phase was oxalic acid solution (0.1%, *v*/*v*), and filtered through a 0.45 µm durapore membrane. The mobile phase flow rate was 1 mL/min. Volume injection was 10 µL and the column temperature was 37 °C. The detection wavelength was 260 nm. The elution time was 10 min. The identification of the ascorbic acid was accomplished by comparing retention time of the standard. Ascorbic acid contents in samples were calculated by regression equation and expressed as g/100 g DW.

### 2.9. Determination of Total Phenol Contents (TPC)

Total phenol contents of samples were determined using Folin-Ciocalteu method according to Rambaran et al. [24], with minor modifications. Briefly, supernatant of red raspberry juice was firstly tenfold diluted in distilled water (1:9, *v*/*v*). Diluted samples (1 mL) were mixed with 4 mL of sodium carbonate solution (7.5%, *w*/*v*) and 5 mL of 10% (*v*/*v*) Folin-Ciocalteu reagent and were allowed to stand at room temperature for 1 h. The absorbance of mixtures was measured at 765 nm. A calibration curve was obtained using 0–100 µg gallic acid (GAE)/mL and was used to calculate the total phenol contents of samples. Total phenol contents in samples were expressed as g (GAE)/100 g DW.

### 2.10. Statistical Analysis

All biochemical determinations were repeated three times, and they were expressed as the mean ± standard deviation (SD). Duncan’s multiple-comparison test, one-way analysis of variance (ANOVA) was performed by using SPSS 20.0 Statistics (SPSS Inc., Chicago, IL, USA) to identify significant differences (*p* < 0.05) among accessions. Principal component analysis (PCA) and cluster analysis (CA) were performed by using OriginPro 2021b Statistics (OriginLab Inc., Northampton, MA, USA).

## 3. Results and Discussion

### 3.1. Length, Diameter, Weight and Appearance Range of Red Raspberry

Table 1 shows the physical properties of 24 red raspberry varieties. The values of fruit length, diameter and weight varied from 1.52 ± 0.11 to 2.72 ± 0.12 cm, 1.42 ± 0.17 to 2.43 ± 0.23 cm, and 1.54 ± 0.11 to 4.99 ± 0.21 g, respectively. Among them, ‘DNS5’, ‘Heritage’, ‘Beijing32’, ‘Rerille’ and ‘Nootka’ had significantly higher fruit length, diameter and weight values, while ‘DNS9’ has the lowest length, diameter and weight values. Differences in the fruit size can be explained by a very high variability within individual genotypes [15,25,26]. Liu et al. [27] found that fruit size was affected by several auxin response factors through modulating the genes transcription. Several studies have shown that raspberry yield was positively correlated with fruit size [28,29]. Therefore, ‘DNS5’, ‘Heritage’, ‘Beijing32’, ‘Rerille’ and ‘Nootka’ were inferred to have relatively high yields.

**Figure 1:**
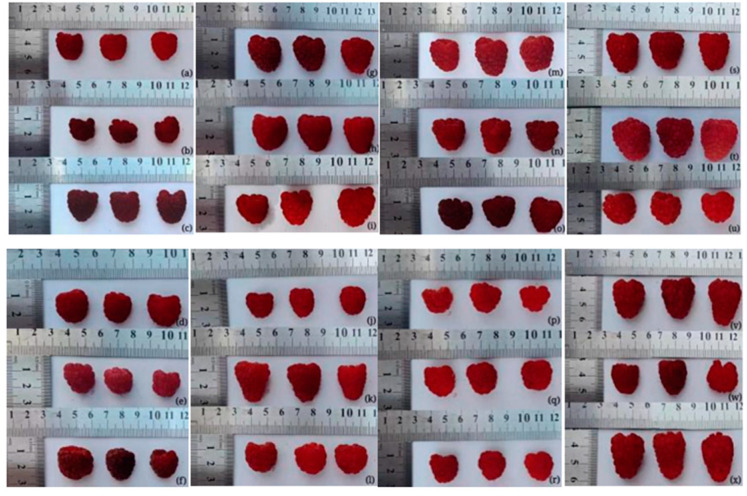
The size and shape of 24 red raspberry varieties. (**a**): ‘European red’, (**b**): ‘Beijing21’, (**c**): ‘Boyne’, (**d**): ‘DNS9’, (**e**): ‘Beijing19’, (**f**): ‘Summit’, (**g**): ‘Beijing32’, (**h**): ‘Beijing10’, (**i**): ‘Nootka’, (**j**): ‘Fertod zamatos’, (**k**): ‘DNS4’, (**l**): ‘DNS2’, (**m**): ‘Samodiva’, (**n**): ‘DNS1’, (**o**): ‘Royalty’, (**p**): ‘Tulameen’, (**q**): ‘Canby’, (**r**): ‘Bulgaskc’, (**s**): ‘Ruby’, (**t**): ‘Heritage’, (**u**): ‘Schopska’, (**v**): ‘DNS5’, (**w**): ‘Willamette’, (**x**): ‘Rerille’.

Most red raspberry were frustoconical-shaped, while ‘European red’, ‘Beijing21’, ‘DNS9’, ‘Beijing19’, ‘DNS2’, ‘Royalty’, ‘Tulameen’, ‘Canby’, ‘Bulgaskc’ and ‘Schopska’ were near-spherical (Figure 1). Harris et al. [30] found that the main factors affecting berry shape, especially near spherical shape, were sufficient carbohydrates and water, due to cell mitotic division and cell expansion by accumulation of water and secondary metabolites [31]. Previously, Stojanov et al. [32] researched vegetative growth, reproductive development, productivity, fruit physical properties, levels of some primary metabolites and active compounds in the ‘Meeker’ red raspberry, and found that the higher the sphericity index, the higher the sweetness. Further research is needed to clarify the notion that red raspberry with a near-spherical shape is sweeter than those of frustoconical-shape.

### 3.2. Moisture, Total Sugar, Reducing Sugar, Titratable Acidity (TAC), Soluble Solids Contents (SSC), pH and Sugar-Acid Ratio (SAR)

The moisture, total sugar, reducing sugar, TAC, SSC, pH and SAR of 24 red raspberry varieties are shown in Table 2. The moisture ranged from 80.73 ± 0.72 to 86.15 ± 1.05 g/100 g FW, with an average of 84.49 g/100 g FW. Moisture content was main attributes in evaluating the quality of fruit. Xie et al. [33] reported that a decrease in moisture contents caused significantly reduced total phenolic contents, total flavonoid contents and antioxidant activities in strawberry; at the same time, the moisture contents of fruit were also related to the contents of soluble solid [34]. The contents of total sugar and reducing sugar varied from 50.78 ± 1.99 to 82.64 ± 0.21 g/100 g DW, and 30.26 ± 0.33 to 55.76 ± 2.66 g/100 g DW, respectively. Among them, ‘Canby’ and ‘Samodiva’ had significantly higher total sugar contents, while ‘Fertod zamatos’ was the lowest (50.78 ± 1.99 g/100 g DW). The ‘Beijing19’, ‘DNS4’ and ‘Royalty’ varieties had significantly higher reducing sugar contents, while ‘European red’ was the lowest (30.26 ± 0.33 g/100 g DW). The contents of TAC, SSC and pH were parameters that influence the ripening and quality of the fruits. The values of TAC, SSC and pH varied from 4.90 ± 1.19 to 17.51 ± 0.51 g/100 g DW, 6.33 ± 0.58 to 14.33 ± 0.58 °Brix, and 2.53 ± 0.02 to 3.19 ± 0.02, respectively. The ‘Beijing10’ was highest in TAC and lowest in SSC. The varieties ‘European red’, ‘DNS9’, ‘Bulgaskc’ and ‘Canby’ were higher in pH and lower in TAC. The moisture, total sugar, reducing sugar and TAC values were similar to data reported by others for the majority of raspberry varieties studied [24,35,36,37], while the SSC and pH values were lower than most of the data determined by others [38,39]. This phenomenon might be due to the strong relationship between fruits acidity and metabolic behavior, since some fruit may consume organic acids during the respiratory process [40].

The values of SAR in this study varied from 2.90 to 11.91. The ‘European red’, ‘Bulgaskc’, ‘Canby’, ‘Samodiva’ and ‘DNS9’ had the higher SAR values of 11.91, 9.39, 9.04, 8.96 and 8.34, respectively, so they tasted sweeter and thus might be suitable for fresh-eating or processing to juice or other products directly. The ‘Beijing10’ had the lowest SAR value, thus it might be suitable for acidophilic consumers or need to adjust the taste in processing for its excessive sour taste. Fruit taste can be scientifically evaluated and classified by TAC, SSC and SAR. According to the three indexes, fruit can be divided into five categories: sweet, sour and sweet, moderate, sweet and sour, sour [41]. Agredano-De La Garza et al. [42] divided Nance into three group, including “very sweet” with SAR value of 40.2, “sweet” with SAR value of 21, “sour” with SAR values of 3.7 to 8.

### 3.3. Individual Sugar and Organic Acid Contents

#### 3.3.1. Individual Sugar Contents

Sugar provides energy for the life activities of human, and was an important fruit sensory quality [43]. Six individual sugars, including l-rhamnose monohydrate, fructose, glucose, sucrose, maltose and d-trehalose anhydrous, were determined and the contents are shown in Table 3. The fructose and glucose were found to be the predominant monosaccharide and sucrose was the major disaccharide in all varieties. The fructose, glucose and sucrose contents were 13.79 ± 0.52 to 37.46 ± 0.28 g/100 g DW, 10.91 ± 0.89 to 30.98 ± 0.65 g/100 g DW, 0.15 ± 0.01 to 38.29 ± 8.55 g/100 g DW, respectively. Among the 24 red raspberry varieties, the ‘Beijing19’, ‘DNS2’, ‘Schopska’ and ‘Beijing21’ had higher level in fructose and glucose contents, while the significant lower level in sucrose contents. Interestingly, ‘DNS9’, ‘Canby’, ‘European red’ and ‘Samodiva’ had significantly higher sucrose contents and lower fructose and glucose contents. Mouillot [44] presented that glucose was perceived to be less intense and pleasant than the fructose and the sucrose, and the fructose was perceived to be less intense and pleasant than the sucrose. The results of Stavang et al. [45] confirmed that the sweetness was significantly positively correlated with concentrations of sucrose (*r* = 0.72, *p* < 0.05). Therefore, ‘DNS9’, ‘Canby’, ‘European red’ and ‘Samodiva’ might have relatively pleasant sweetness than other varieties.

#### 3.3.2. Organic Acid Contents

Organic acids were very important for fruit quality in terms of taste and contributed to the acidity of fruits [46]. The contents of eight organic acids, including oxalic acid, tartaric acid, malic acid, α-ketoglutaric acid, lactic acid, citric acid, fumaric acid and succinic acid, in the 24 red raspberry varieties are shown in Table 4.

The citric acid was the predominant organic acid in all varieties, with the contents from 2.95 ± 0.19 to 13.85 ± 2.38 g/100 g DW, followed by malic acid with the contents from 0.31 ± 0.02 to 2.37 ± 0.26 g/100 g DW. Oxalic acid and α-ketoglutaric acid were detected in all varieties with minor contents, while tartaric acid, lactic acid, fumaric acid and succinic acid existed in some varieties. Contents of fumaric acid were relatively low with values of 0.01 or 0.02 g/100 g DW. Citric acid was also identified as the major organic acids in wild red raspberry accessions from northern Turkey, in which small amounts of malic acids were also detected [16]. Mortazavi and Al-Farsi et al. [47,48] reported that organic acid contents varied according to variety and stage of maturity, and these organic acids may inhibit microorganisms growth and hence affect the preserving of fruits. The antimicrobial activity of organic acids was thought to be due to their ability to freely cross over the cell membrane and then dissociate into a proton and a corresponding ion, which leads to the increase in intracellular acidity and accelerates the metabolic disorders of the cells [49]. In addition, these organic acids enhanced appetite and facilitated digestion and stabilization of the water-soluble vitamins B and C and improved potassium, copper, zinc, iron and calcium absorption [50]. Among the 24 red raspberry varieties, the ‘Heritage’ was highest in citric acid, malic acid and TAC contents, and the lowest in pH. The ‘Beijing10’ was highest in citric acid and TAC contents, and the lowest in SAR and sucrose contents. Therefore ‘Heritage’ and ‘Beijing10’ might have a sharper acidity taste. The ‘European red’, ‘DNS9’ and ‘Bulgaskc’ had significantly lower citric acid contents and higher SAR and sucrose contents, inferring with relatively high sweetness taste.

### 3.4. Sensory Characteristics Analysis

**Figure 2:**
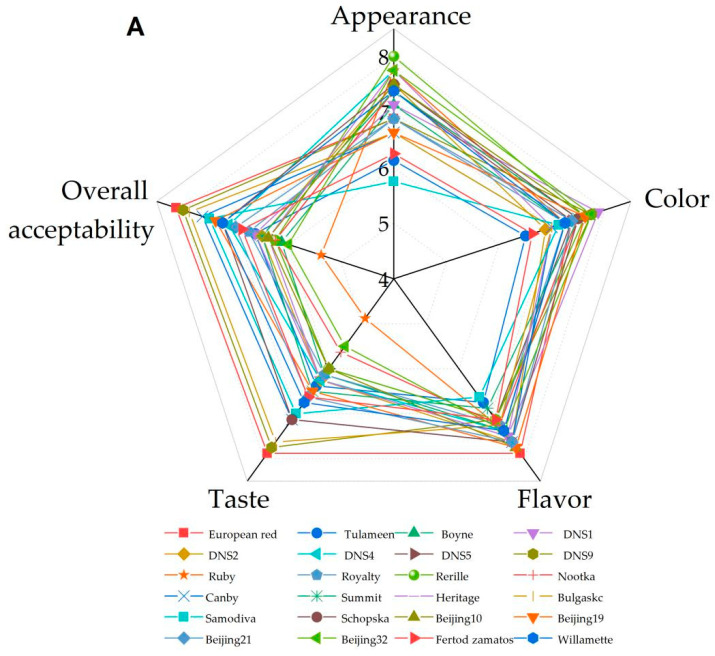

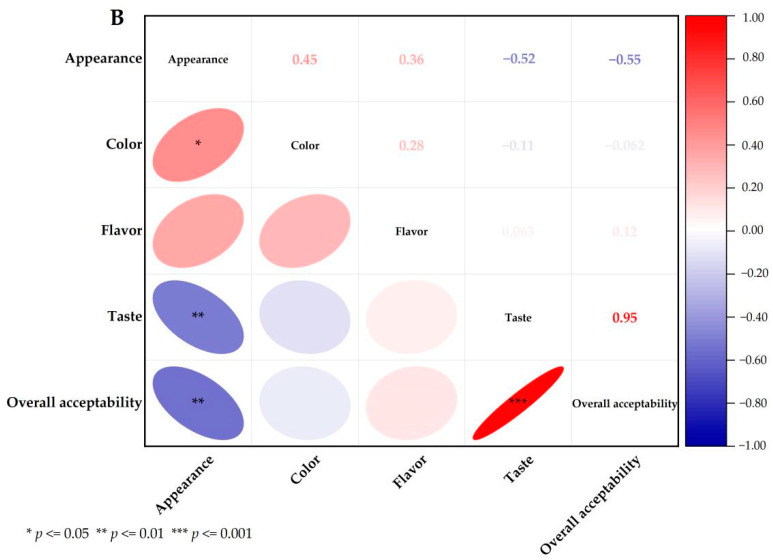
Radar plot showing sensory characteristics evaluation of 24 red raspberry varieties (**A**) and correlation analysis of five sensory characteristics (**B**).

The sensory characteristics attributes, including appearance, color, flavor, taste and overall acceptability, and were presented in Figure 2A. The ‘Rerille’, ‘DNS4’, ‘Ruby’, ‘Heritage’ and ‘Beijing32’ displayed higher values than other varieties in appearance, while ‘DNS1’, ‘DNS9’, ‘Beijing32’, ‘Rerille’, ‘Beijing10’ and ‘Beijing19’ showed much higher value than other varieties in color. The ‘European red’, ‘Beijing10’, ‘Beijing19’, ‘DNS2’, ‘Canby’, ‘Schopska’ and ‘Beijing21’ displayed higher values than the other varieties in flavor, while ‘European red’, ‘DNS9’ and ‘Bulgaskc’ showed much higher value than other varieties in taste and overall acceptability. The ‘European red’, ‘DNS9’ and ‘Bulgaskc’ were lower in citric acid contents, but higher in SAR; therefore, they were more acceptable for fresh eating.

The correlation of five sensory characteristics (appearance, color, flavor, taste and overall acceptability) are shown in Figure 2B. It was observed that the taste was well correlated to the overall acceptability (*r* = 0.95, *p* ≤ 0.001). The appearance had significantly negative correlation with the taste and overall acceptability, with the correlation coefficients of −0.52 and −0.55 (*p* ≤ 0.01), respectively. These findings indicated that consumers tend to have a higher overall acceptability for fruits that taste better, and their vision may be affected by the attractiveness of fruit, while ignoring the smell of fruit. Considering these factors, future sensory evaluation should strengthen the potential sensory bias caused by flavor preference.

### 3.5. Ascorbic Acid Contents

**Figure 3:**
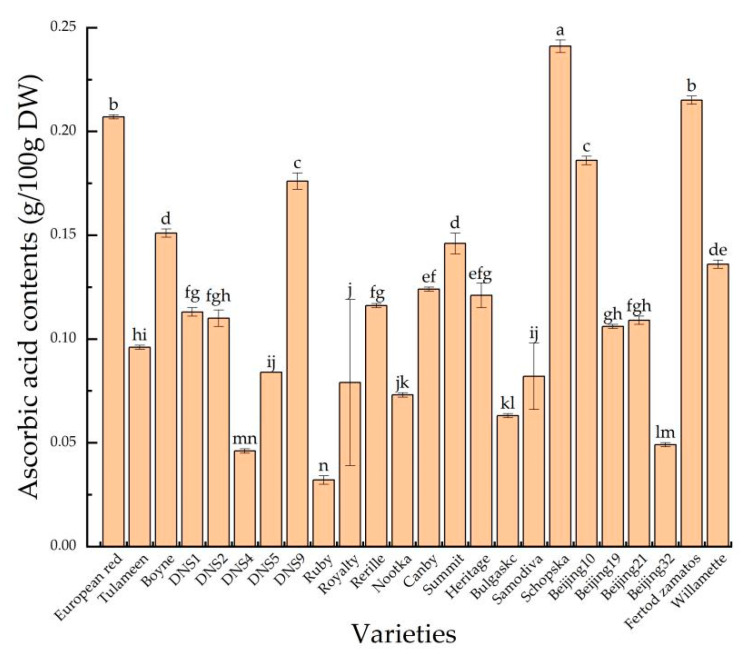
The ascorbic acid contents of 24 red raspberry varieties. Values are given as the mean ± standard deviation (*n* = 3), and the different letters were significantly different (*p* < 0.05).

Ascorbic acid contents are one of the most important compositional parameters determining the nutritional and health related properties of plant foods [51]. The results of the present study (Figure 3) revealed differences in the ascorbic acid concentrations between the studied varieties. According to our data, the average ascorbic acid contents of the red raspberry was 0.12 g/100 g DW. The ascorbic acid contents ranged from 0.03 to 0.24 g/100 g DW with ‘Schopska’ holding the highest content, yet ‘Ruby’ having the lowest content. Ascorbic acid has an important physiological role in numerous metabolic functions including tissue growth and maintenance, amelioration of oxidative stress, and immune regulation [52,53]. Van de Velde et al. revealed that ascorbic acid in red raspberry was associated with antioxidant effects, and the higher the concentration of ascorbic acid, the more powerful the ability to provide electrons or hydrogen atoms. A strong capacity of ascorbic acid to donate electrons or hydrogen atoms and then act as antioxidants to reduce the oxidative damage caused by reactive oxygen species (ROS) was also shown [54].

### 3.6. Total Phenol Contents (TPC)

**Figure 4:**
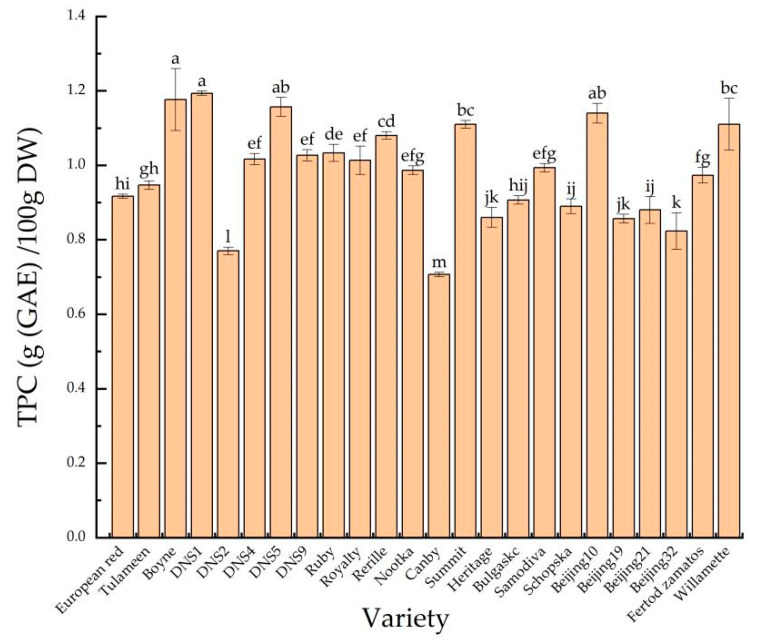
The total phenol contents (TPC) of 24 red raspberry varieties. Values are given as the mean ± standard deviation (*n* = 3), and the different letters were significantly different (*p* < 0.05).

Researchers revealed that berry fruits are great dietary sources of phenols. Phenol may act as strong antioxidants and thus could help in the prevention of many diseases [6]. The contents of total phenol in the 24 red raspberry varieties (Figure 4) ranged from 0.77 to 1.19 g/100 g DW. The ‘DNS1’, ‘Boyne’, ‘DNS5’, ‘Beijing10’ varieties had the highest phenol contents indicating that they might exert stronger antioxidant abilities. The results clarified the differences in TPC among the 24 red raspberry varieties.

Schulz et al. [55] detected that the phenolic compounds in red raspberry were flavonols, ellagitannins, phenolic acids. Ellagic acid, caffeic acid, catechin, gallic acid, epicatechin, quercetin, and kaempferol were the main phenolics found in red raspberry. Moreover, phenols from the fruit have been shown to have rich biological activities involving anticarcinogenic [56], antimutagenic [57], antibacterial [58], antioxidant, and antiradical properties [59], which were evident from various in vitro and animal model studies. In addition, some phenolics compounds, such as ferulic acid, caffeic acid and p-coumaric acid, have been described as functionally important compounds with good antioxidant activity [60]. Further research need be carried out to identify the details of phenolic compounds in this study.

### 3.7. Principal Component Analysis (PCA)

**Figure 5:**
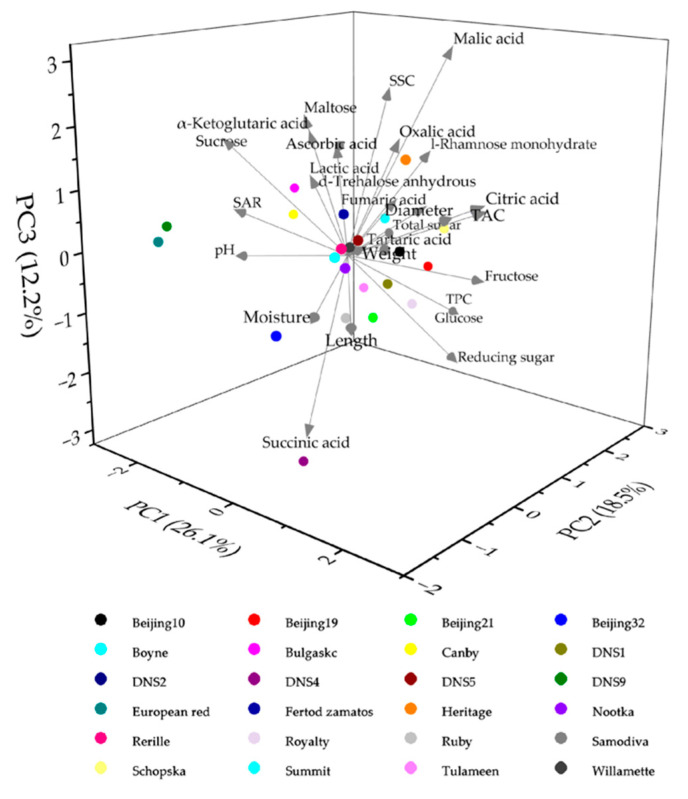
Three-dimensional principal component analysis (PCA) plot of the quantitative traits and 24 red raspberry varieties.

PCA is used to identify potential trait combinations [61]. It extracts principal components based on the characteristics of each eigenvalue. A total of 7 principal components (PCs) were identified (Table S1) and contributed to the total variation of 83.567%, replacing most of the information of the original variables [62]. In addition, a three-dimensional PCA plot was constructed based on the first three components (Figure 5).

As reported in Table S1, the loading of variables showed that primarily sucrose, citric acid, TAC, SAR, pH, length, diameter and weight contents took part in the formation of PC1 (variance contribution rate of 26.06%). The variance contribution rate of PC2 was 18.47%, which was primarily responsible for the differences in the contents of l-rhamnose monohydrate, fructose, glucose, total sugar, reducing sugar, SSC, moisture and TPC. PC3 accounted for 12.16% of the variance contribution rate, and was related to the contents of malic acid, succinic acid, reducing sugar and SSC contents. PCA results showed that TAC, SAR, pH, length, diameter, weight, sucrose and citric acid were the most important property indexes for processing attributes in the evaluating process.

The comprehensive evaluation indexes were the accumulated sum of the factors’ score of each sample and the weight value of each principal component [63]. Therefore, PC1-PC7 were selected as the main components of comprehensive quality. PC1 was selected as the main component of fresh utilization. The comprehensive quality score and fresh utilization score in the evaluation index values were −0.606 to 0.883, and −0.294 to 0.581, respectively (Table S2).

Comprehensive quality is a concept not only based on the single quality attributes but also on interactions among them. It includes the external characteristics (weight, shape, and size), taste qualities (SSC, TAC, and SAR), and also involves nutritional value (ascorbic acid and TPC) et al. [64]. According to the breeding characteristics and current market demand, red raspberry was usually consumed as fresh fruit or processed fruit products. A good fruit germplasm for fresh market consumption requires high SSC and SAR as well as high fruit weight [65,66]. The top five red raspberry varieties were ‘European red’, ‘DNS9’, ‘DNS4’, ‘Canby’ and ‘Beijing32’ based on the final rank of comprehensive quality. For fresh utilization, the top five red raspberry varieties were ‘DNS9’, ‘European red’, ‘Bulgaskc’, ‘Canby’ and ‘Samodiva’. The varieties ‘DNS5’, ‘Heritage’, ‘Beijing10’, ‘Schopska’ and ‘Rerille’ were ranked low for both comprehensive quality score and fresh utilization; they had significantly higher fruit length, diameter, weight, TAC values and lower in pH and SAR, indicating that these five kinds of red raspberries were characterized by large fruit size and sour taste. Therefore, it was inferred that red raspberry with low-ranking values were not suitable for fresh-eating and need to adjust the taste in processing for their excessive sour taste, but could be used in future breeding programs.

### 3.8. Cluster Analysis (CA)

**Figure 6:**
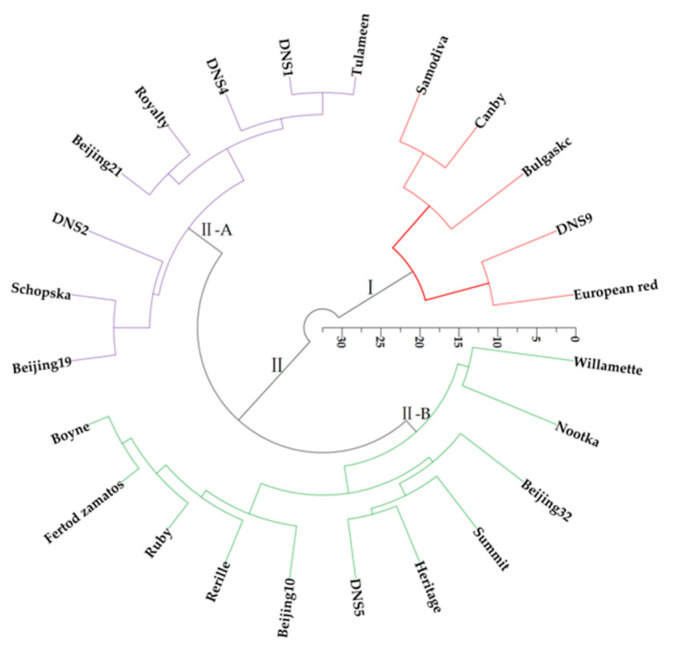
Cluster analysis of individual sugar, organic acids and SAR in the 24 red raspberry varieties.

The PCA results showed that TAC, SAR, pH, length, diameter, weight, sucrose and citric acid were the most important property indexes for processing attributes in the evaluating process. Although red raspberry comprehensive quality was closely related to these property indexes, the taste was affected by individual sugar, organic acids and SAR [44,46,67]. The cluster analysis based on individual sugar, organic acids and SAR clustered the 24 red raspberry varieties into two major clusters (Figure 6).

The ‘European red’, ‘DNS9’, ‘Bulgaskc’, ‘Canby’ and ‘Samodiva’ were placed into the first cluster (Ⅰ), and the rest of the red raspberry varieties was placed into the second cluster (Ⅱ). Ⅱ-A was comprised of 8 red raspberry varieties, Ⅱ-B included a total of 11 red raspberry varieties. In cluster (Ⅰ), ‘European red’, ‘DNS9’, ‘Bulgaskc’, ‘Canby’ and ‘Samodiva’ were grouped together because they had higher values of sucrose and SAR, and lower values of citric acid. In cluster (Ⅱ), Ⅱ-A was the red raspberry with higher fructose and glucose values, and Ⅱ-B was composed of other remaining varieties.

## 4. Conclusions

Overall, significant diversity was revealed regarding the fruit quality and nutritional properties of the evaluated varieties by determining the physicochemical properties, bioactive compounds and sensory characteristics, and evaluating the comprehensive quality between different varieties based on the analysis of PCA and CA.

According to PCA, the top five red raspberry varieties based on the final rank of comprehensive quality were ‘European red’, ‘DNS9’, ‘DNS4’, ‘Canby’ and ‘Beijing32’, and the top five red raspberry varieties for fresh utilization were ‘DNS9’, ‘European red’, ‘Bulgaskc’, ‘Canby’ and ‘Samodiva’. The second group of PCA was consistent with the results of CA for fresh-eating or processing to juice or other products directly, because they had suitable SAR, however, other varieties with relatively low SAR were unsuitable for fresh eating and need to adjust their excessive sour taste during processing.

The ‘DNS4’ and ‘Beijing32’ had better appearance and bigger fruits, ‘European red’, ‘Schopska’ and ‘Fertod zamatos’ had higher contents of ascorbic acid. The ‘Boyene’, ‘DNS1’, ‘DNS5’ and ‘Beijing10’ contained more TPC, and these varieties might release more commercial potential abilities in the future.

## Figures and Tables

**Table 1:** The physical properties of 24 red raspberry varieties.

Varieties	Pedigree	Length/cm	Diameter/cm	Weight/g
European red	Russia	1.84 ± 0.21 ^f^^–^^i^	1.58 ± 0.21 ^f,g^	2.38 ± 0.21 ^f,g^
Tulameen	Canada	1.58 ± 0.14 ^h,i^	1.61 ± 0.21 ^e^^–^^g^	2.16 ± 0.23 ^g,h^
Boyne	Canada	1.86 ± 0.27 ^f^^–^^i^	2.01 ± 0.31 ^b^^–^^d^	2.37 ± 0.11 ^f,g^
DNS1	Northeast Agricultural University	2.02 ± 0.25 ^d^^–^^f^	2.05 ± 0.11 ^a^^–^^d^	2.94 ± 0.14 ^d^
DNS2	Northeast Agricultural University	1.77 ± 0.25 ^f^^–^^i^	2.03 ± 0.13 ^b^^–^^d^	2.84 ± 0.17 ^d,e^
DNS4	Northeast Agricultural University	2.72 ± 0.12 ^a^	1.97 ± 0.18 ^b^^–^^f^	2.50 ± 0.19 ^e^^–^^g^
DNS5	Northeast Agricultural University	2.60 ± 0.12 ^a,b^	2.34 ± 0.12 ^a,b^	4.99 ± 0.21 ^a^
DNS9	Northeast Agricultural University	1.52 ± 0.11 ^i^	1.42 ± 0.17 ^g^	1.54 ± 0.11 ^i^
Ruby	The United States	2.34 ± 0.21 ^b^^–^^d^	1.95 ± 0.11 ^b^^–^^f^	4.02 ± 0.21 ^b,c^
Royalty	The United States	1.98 ± 0.21 ^e^^–^^g^	2.03 ± 0.23 ^b^^–^^d^	2.97 ± 0.26 ^d^
Rerille	The United States	2.54 ± 0.21 ^a^^–^^c^	2.24 ± 0.26 ^a^^–^^c^	3.90 ± 0.41 ^c^
Nootka	The United States	2.44 ± 0.27 ^a^^–^^c^	2.18 ± 0.11 ^a^^–^^d^	3.83 ± 0.29 ^c^
Canby	The United States	1.78 ± 0.31 ^f^^–^^i^	1.86 ± 0.14 ^c^^–^^f^	1.95 ± 0.23 ^h,i^
Summit	The United States	1.92 ± 0.11 ^e^^–^^h^	1.99 ± 0.11 ^b^^–^^e^	2.77 ± 0.25 ^d^^–^^f^
Heritage	The United States	2.52 ± 0.11 ^a^^–^^c^	2.43 ± 0.23 ^a^	4.36 ± 0.31 ^b^
Bulgaskc	Bulgaria	1.76 ± 0.11 ^f^^–^^i^	1.78 ± 0.31 ^d^^–^^g^	1.90 ± 0.22 ^h,i^
Samodiva	Bulgaria	1.78 ± 0.16 ^f^^–^^i^	1.80 ± 0.27^d^^–^^f^	2.15 ± 0.19 ^g,h^
Schopska	Bulgaria	1.98 ± 0.11 ^e^^–^^g^	2.14 ± 0.21 ^a^^–^^d^	3.10 ± 0.23 ^d^
Beijing10	Beijing Academy of Agriculture and Forestry Sciences	2.23 ± 0.28 ^c^^–^^e^	2.02 ± 0.20 ^b^^–^^d^	2.85 ± 0.24 ^d,e^
Beijing19	Beijing Academy of Agriculture and Forestry Sciences	1.54 ± 0.11 ^i^	1.80 ± 0.20 ^d^^–^^f^	1.91 ± 0.21 ^h,i^
Beijing21	Beijing Academy of Agriculture and Forestry Sciences	1.62 ± 0.24 ^g^^–^^i^	1.82 ± 0.11 ^d^^–^^f^	1.79 ± 0.18 ^h,i^
Beijing32	Beijing Academy of Agriculture and Forestry Sciences	2.43 ± 0.12 ^a^^–^^c^	2.05 ± 0.27 ^a^^–^^d^	4.17 ± 0.13 ^b,c^
Fertod zamatos	Hungary	1.63 ± 0.12 ^g^^–^^i^	2.08 ± 0.30 ^a^^–^^d^	1.71 ± 0.21 ^i^
Willamette	France	2.04 ± 0.12 ^d^^–^^f^	2.11 ± 0.11 ^a^^–^^d^	2.75 ± 0.27 ^d^^–^^f^

Values are given as the mean ± standard deviation (*n* = 50), and the different letters within each column were significantly different (*p* < 0.05).

**Table 2:** The moisture, total sugar, reducing sugar, titratable acidity (TAC), soluble solids contents (SSC), pH and sugar-acid ratio (SAR) of 24 red raspberry varieties.

Varieties	Moisture (g/100 g FW)	Total Sugar (g/100 g DW)	Reducing Sugar (g/100 g DW)	TAC (g/100 g DW)	SSC (°Brix)	pH	SAR
European red	84.00 ± 0.45 ^c–h,^	58.05 ± 0.81 ^h–j^	30.26 ± 0.33 ^m^	4.90 ± 1.19 ^l^	9.33 ± 0.58 ^d,e^	3.19 ± 0.02 ^a^	11.91
Tulameen	83.45 ± 1.58 ^f–h^	71.75 ± 0.18 ^c,d^	51.87 ± 1.38 ^b^	10.59 ± 1.20 ^h,i^	9.33 ± 0.58 ^d,e^	2.76 ± 0.05 ^c,d^	5.32
Boyne	85.38 ± 0.73 ^a–d^	59.27 ± 0.62 ^g–i^	43.06 ± 2.18 ^f–h^	14.93 ± 0.20 ^b,c^	10.67 ± 0.58 ^c^	2.62 ± 0.05 ^d–g^	4.89
DNS1	86.15 ± 1.05 ^a^	71.70 ± 1.30 ^c,d^	47.77 ± 0.86 ^c^	11.95 ± 3.10 ^f–h^	7.33 ± 0.58 ^g,h^	2.75 ± 0.06^c–e^	4.42
DNS2	80.73 ± 0.72 ^i^	76.36 ± 0.06 ^b^	52.51 ± 0.71 ^b^	14.65 ± 0.37 ^c,d^	12.33 ± 0.58 ^b^	2.61 ± 0.06 ^d–g^	4.37
DNS4	85.16 ± 0.43 ^a–e^	64.28 ± 0.62 ^e,f^	55.69 ± 4.18 ^a^	9.16 ± 0.56 ^i,j^	6.33 ± 0.58 ^h^	2.95 ± 0.13 ^b^	6.46
DNS5	86.13 ± 1.43 ^a^	54.27 ± 2.19 ^j,k^	45.89 ± 1.68 ^c–f^	14.84 ± 0.43 ^c,d^	8.00 ± 0.00 ^f,g^	2.55 ± 0.10^g^	3.89
DNS9	83.88 ± 0.86 ^d–h^	68.74 ± 1.37 ^d^	36.00 ± 2.26 ^k,l^	6.47 ± 0.10 ^k,l^	8.67 ± 0.58 ^e,f^	3.09 ± 0.09 ^a,b^	8.34
Ruby	85.99 ± 0.30 ^a,b^	64.40 ± 0.29 ^e,f^	42.00 ± 3.54 ^g–i^	12.85 ± 2.97 ^d–f^	7.67 ± 0.58 ^f,g^	2.62 ± 0.06 ^d–g^	4.26
Royalty	84.35 ± 0.30 ^b–h^	75.54 ± 0.38 ^b,c^	54.78 ± 0.44 ^a,b^	14.86 ± 0.19 ^c,d^	8.33 ± 0.58 ^e–g^	2.66 ± 0.06 ^c–g^	3.58
Rerille	85.67 ± 1.65 ^a–c^	56.08 ± 0.76 ^i,j^	39.38 ± 0.14 ^i,j^	14.36 ± 0.81 ^c–e^	8.67 ± 0.58 ^e,f^	2.59 ± 0.09 ^f,g^	4.22
Nootka	86.00 ± 0.65 ^a,b^	72.11 ± 1.56 ^c,d^	47.91 ± 0.75 ^c^	14.29 ± 0.52 ^c–e^	7.33 ± 0.58 ^g,h^	2.63 ± 0.03 ^d–g^	3.67
Canby	83.10 ± 0.81 ^g,h^	82.64 ± 0.21 ^a^	43.48 ± 0.82 ^e–h^	7.42 ± 0.55 ^j,k^	11.33 ± 0.58 ^c^	3.16 ± 0.03 ^a^	9.04
Summit	83.59 ± 0.95 ^e–h^	61.56 ± 0.14 ^f–h^	46.6 ± 3.29 ^c–e^	15.46 ± 0.70 ^b,c^	10.67 ± 0.58 ^c^	2.58 ± 0.12 ^f,g^	4.21
Heritage	85.84 ± 0.51 ^a,b^	67.84 ± 0.70 ^d,e^	41.64 ± 0.64 ^g–i^	17.51 ± 0.51 ^a^	11.33 ± 0.58 ^c^	2.55 ± 0.10 ^f,g^	4.57
Bulgaskc	82.90 ± 0.27 ^g,h^	74.79 ± 6.57 ^b,c^	41.30 ± 0.80 ^h,i^	6.44 ± 0.26 ^k,l^	10.33 ± 0.58 ^c,d^	3.18 ± 0.04 ^a^	9.39
Samodiva	85.09 ± 0.66 ^a–f^	82.49 ± 0.87 ^a^	46.30 ± 1.27 ^c–f^	10.72 ± 0.29 ^g–i^	14.33 ± 0.58 ^a^	2.98 ± 0.18 ^b^	8.96
Schopska	82.79 ± 0.46 ^h^	77.12 ± 7.99 ^b^	44.83 ± 0.35 ^c–g^	15.24 ± 1.52 ^b,c^	9.33 ± 0.58 ^d,e^	2.61 ± 0.01 ^d–g^	3.42
Beijing10	85.02 ± 0.76 ^a–f^	63.33 ± 0.53 ^f,g^	43.78 ± 1.66 ^d–h^	16.87 ± 0.38 ^a,b^	7.33 ± 0.58 ^g,h^	2.53 ± 0.02 ^g^	2.90
Beijing19	84.46 ± 0.68 ^a–h^	69.56 ± 1.33 ^d^	55.76 ± 2.66 ^a^	12.57 ± 0.32 ^e–g^	13.00 ± 0.00 ^b^	2.71 ± 0.01 ^c–f^	6.66
Beijing21	84.50 ± 1.21 ^a–g^	71.96 ± 0.39 ^c,d^	46.89 ± 0.59 ^c,d^	10.50 ± 1.05 ^h,i^	7.33 ± 0.58 ^g,h^	2.80 ± 0.08 ^c^	4.51
Beijing32	85.99 ± 0.78 ^a,b^	60.39 ± 1.42 ^f–h^	38.73 ± 2.12 ^i–k^	14.35 ± 0.36 ^c–e^	7.33 ± 0.58 ^g,h^	2.58 ± 0.13 ^f,g^	3.65
Fertod zamatos	83.60 ± 0.94 ^e–h^	50.78 ± 1.99 ^k^	36.29 ± 1.21 ^j–l^	15.23 ± 0.18 ^b,c^	9.33 ± 0.58 ^d,e^	2.58 ± 0.01 ^f,g^	3.73
Willamette	83.97 ± 0.80 ^d–h^	55.18 ± 1.34 ^i,j^	33.78 ± 1.19 ^l^	14.03 ± 0.27 ^c–e^	10.33 ± 0.58 ^c,d^	2.59 ± 0.11 ^e–g^	4.59

Values are given as the mean ± standard deviation (*n* = 3), and the different letters within each column were significantly different (*p* < 0.05).

**Table 3:** Individual sugar contents of 24 red raspberry varieties.

Varieties	l-Rhamnose Monohydrate (g/100 g DW)	Fructose (g/100 g DW)	Glucose (g/100 g DW)	Sucrose (g/100 g DW)	Maltose (g/100 g DW)	d-Trehalose Anhydrous (g/100 g DW)
European red	0.54 ± 0.02 ^h,i^	13.79 ± 0.52 ^l^	10.91 ± 0.89 ^k^	31.79 ± 0.73 ^b,c^	0.33 ± 0.20 ^d–h^	0.19 ± 0.01 ^b,c^
Tulameen	0.84 ± 0.28 ^c–h^	26.54 ± 1.14 ^f^	27.11 ± 2.49 ^c^	0.29 ± 0.03 ^j^	0.30 ± 0.10 ^e–i^	0.20 ± 0.00 ^b,c^
Boyne	0.88 ± 0.50 ^c–h^	19.61 ± 2.53 ^k^	17.07 ± 2.72 ^g,h^	2.32 ± 0.35 ^i,j^	0.32 ± 0.09 ^d–h^	0.07 ± 0.13 ^d^
DNS1	1.24 ± 0.33 ^b,c^	25.83 ± 0.55 ^f,g^	27.62 ± 0.53 ^c^	0.33 ± 0.05 ^j^	0.26 ± 0.21 ^f–j^	0.26 ± 0.05 ^b^
DNS2	1.77 ± 0.06 ^a^	32.98 ± 0.55 ^c,d^	30.98 ± 0.65 ^a^	9.89 ± 0.08 ^f^	0.23 ± 0.07 ^f–j^	0.18 ± 0.00 ^b,c^
DNS4	0.32 ± 0.01 ^i^	23.88 ± 0.04 ^h,i^	27.24 ± 0.03 ^c^	2.32 ± 0.00 ^i,j^	0.05 ± 0.01 ^i,j^	0.21 ± 0.00 ^b,c^
DNS5	1.22 ± 0.18 ^b,c^	23.77 ± 1.20 ^h,i^	18.22 ± 1.04 ^e–g^	6.59 ± 0.62 ^g,h^	0.42 ± 0.18 ^d–f^	0.07 ± 0.13 ^d^
DNS9	0.72 ± 0.29 ^e–h^	14.43 ± 2.89 ^l^	13.26 ± 3.09 ^i,j^	38.29 ± 8.55 ^a^	0.35 ± 0.18 ^d–h^	0.13 ± 0.12 ^c,d^
Ruby	0.85 ± 0.08 ^c–h^	21.91 ± 0.07 ^j^	18.99 ± 0.38 ^e–g^	0.16 ± 0.04 ^j^	0.10 ± 0.04 ^h–j^	0.22 ± 0.01 ^b,c^
Royalty	0.47 ± 0.01 ^h,i^	29.39 ± 0.91 ^e^	27.15 ± 1.10 ^c^	2.17 ± 0.14 ^i,j^	0.30 ± 0.08 ^e–h^	0.19 ± 0.00 ^b,c^
Rerille	0.78 ± 0.02 ^d–h^	22.32 ± 0.52 ^i,j^	15.30 ± 0.38 ^h,i^	5.34 ± 0.48 ^h,i^	0.39 ± 0.02 ^d–g^	0.22 ± 0.01 ^b,c^
Nootka	0.73 ± 0.00 ^e–h^	29.42 ± 0.07 ^e^	12.03 ± 0.58 ^j,k^	13.59 ± 0.12 ^e^	0.15 ± 0.02 ^g–j^	0.22 ± 0.00 ^b,c^
Canby	0.87 ± 0.07 ^c–h^	24.78 ± 0.33 ^f–h^	22.86 ± 0.13 ^d^	33.09 ± 0.32 ^b^	0.84 ± 0.06 ^ab^	0.19 ± 0.02 ^b,c^
Summit	1.11 ± 0.03 ^b–e^	26.51 ± 0.43 ^f^	23.72 ± 0.40 ^d^	9.48 ± 1.04 ^f,g^	0.33 ± 0.27 ^d–h^	0.21 ± 0.02 ^b,c^
Heritage	0.72 ± 0.01 ^e–h^	21.16 ± 0.15 ^j,k^	19.88 ± 0.11 ^e^	12.17 ± 0.06 ^e,f^	0.75 ± 0.02 ^bc^	0.45 ± 0.02 ^a^
Bulgaskc	1.43 ± 0.67 ^a,b^	25.11 ± 0.50 ^f–h^	23.13 ± 0.32 ^d^	20.22 ± 0.05 ^d^	0.98 ± 0.03 ^a^	0.20 ± 0.01 ^b,c^
Samodiva	1.21 ± 0.07 ^b,c^	24.59 ± 0.95 ^g,h^	22.88 ± 0.92 ^d^	28.79 ± 2.31 ^c^	0.55 ± 0.17 ^c–e^	0.23 ± 0.02 ^b,c^
Schopska	0.98 ± 0.05 ^c–g^	35.79 ± 0.80 ^b^	27.87 ± 0.16 ^b,c^	2.16 ± 0.24 ^i,j^	0.26 ± 0.12 ^f–j^	0.19 ± 0.02 ^b,c^
Beijing10	1.20 ± 0.01 ^b,c^	25.81 ± 0.06 ^f,g^	17.55 ± 0.12 ^f,g^	0.15 ± 0.01 ^j^	0.03 ± 0.00 ^j^	0.23 ± 0.00 ^b,c^
Beijing19	1.17 ± 0.23 ^b–d^	37.46 ± 0.28 ^a^	29.85 ± 0.13 ^a,b^	0.15 ± 0.03 ^j^	0.24 ± 0.12 ^f–j^	0.21 ± 0.02 ^b,c^
Beijing21	1.06 ± 0.01 ^b–f^	31.52 ± 0.10 ^d^	28.61 ± 0.05 ^b,c^	0.35 ± 0.00 ^j^	0.13 ± 0.00 ^g–j^	0.22 ± 0.00 ^b,c^
Beijing32	0.88 ± 0.06 ^c–h^	20.89 ± 1.25 ^j,k^	13.96 ± 1.02 ^i,j^	13.31 ± 0.45 ^e^	0.57 ± 0.33 ^c,d^	0.15 ± 0.13 ^c,d^
Fertod zamatos	0.69 ± 0.01 ^f–i^	19.85 ± 0.08 ^k^	19.50 ± 0.11 ^e,f^	2.14 ± 0.03 ^i,j^	0.19 ± 0.01 ^f–j^	0.26 ± 0.01 ^b^
Willamette	0.64 ± 0.00 ^g–i^	34.00 ± 0.05 ^c^	12.09 ± 2.32 ^j,k^	2.23 ± 0.19 ^i,j^	0.18 ± 0.01 ^f–j^	0.19 ± 0.00 ^b,c^

Values are given as the mean ± standard deviation (*n* = 3), and the different letters within each column were significantly different (*p* < 0.05).

**Table 4:** Organic acid contents of 24 red raspberry varieties.

Varieties	Oxalic Acid (g/100 g DW)	Tartaric Acid (g/100 g DW)	Malic Acid (g/100 g DW)	α-Ketoglutaric Acid (g/100 g DW)	Lactic Acid (g/100 g DW)	Citric Acid (g/100 g DW)	Fumaric Acid (g/100 g DW)	Succinic Acid (g/100 g DW)
European red	0.13 ± 0.01 ^g^	0.28 ± 0.02 ^e–g^	1.06 ± 0.15 ^i^	0.06 ± 0.01 ^k,l^	n.d.	2.97 ± 0.02 ^l^	0.01 ± 0.01 ^c^	n.d.
Tulameen	0.13 ± 0.00 ^g^	0.29 ± 0.02 ^e,f^	1.23 ± 0.06 ^f–i^	0.13 ± 0.01 ^e^	0.40 ± 0.05 ^d,e^	7.36 ± 0.41 ^h,i^	0.01 ± 0.00 ^b^	n.d.
Boyne	0.19 ± 0.04 ^c,d^	n.d.	1.23 ± 0.14 ^f–i^	0.06 ± 0.00 ^j,k^	0.20 ± 0.02 ^h,i^	11.69 ± 0.11 ^c,d^	0.01 ± 0.00 ^b^	n.d.
DNS1	0.19 ± 0.00 ^c,d^	0.26 ± 0.02 ^f,g^	1.26 ± 0.12 ^f–i^	0.14 ± 0.01 ^e^	0.21 ± 0.01 ^h,i^	9.21 ± 0.22 ^f,g^	0.01 ± 0.00 ^b^	n.d.
DNS2	0.21 ± 0.01 ^b^	n.d.	1.72 ± 0.02 ^c^	0.08 ± 0.02 ^g–j^	0.17 ± 0.02 ^i^	12.27 ± 0.22 ^c^	0.02 ± 0.00 ^a^	n.d.
DNS4	0.14 ± 0.01 ^f,g^	0.19 ± 0.01 ^h^	0.31 ± 0.02 ^k^	0.04 ± 0.00 ^l^	0.27 ± 0.01 ^g,h^	4.63 ± 0.19 ^j,k^	0.01 ± 0.00 ^b^	0.33 ± 0.06 ^a^
DNS5	0.21 ± 0.01 ^b,c^	0.37 ± 0.01 ^c,d^	1.62 ± 0.28 ^c–e^	0.21 ± 0.02 ^c^	0.49 ± 0.01 ^c^	9.86 ± 1.18 ^e,f^	0.01 ± 0.00 ^b^	n.d.
DNS9	0.14 ± 0.01 ^f,g^	0.19 ± 0.01 ^h^	0.71 ± 0.07 ^j^	0.25 ± 0.01 ^b^	0.74 ± 0.02 ^a^	3.81 ± 0.33 ^k^	n.d.	n.d.
Ruby	0.19 ± 0.00 ^c,d^	0.27 ± 0.01 ^f,g^	1.22 ± 0.00 ^g–i^	0.06 ± 0.00 ^j,k^	n.d.	9.43 ± 0.01 ^f^	0.01 ± 0.00 ^b^	n.d.
Royalty	0.17 ± 0.01 ^d,e^	0.35 ± 0.06 ^d^	1.51 ± 0.23 ^c–g^	0.05 ± 0.02 ^k,l^	0.31 ± 0.19 ^f,g^	11.13 ± 1.31 ^c,d^	0.01 ± 0.00 ^b^	n.d.
Rerille	0.29 ± 0.01 ^a^	0.41 ± 0.07 ^c^	1.56 ± 0.18 ^c–e^	0.06 ± 0.02 ^i–k^	0.33 ± 0.11 ^e–g^	10.61 ± 0.65 ^d,e^	0.01 ± 0.00 ^b^	0.08 ± 0.04 ^c^
Nootka	0.22 ± 0.01 ^b^	n.d.	1.81 ± 0.01 ^b,c^	0.09 ± 0.01 ^f,g^	0.35 ± 0.03 ^d–g^	11.33 ± 0.02 ^c,d^	n.d.	n.d.
Canby	0.16 ± 0.01 ^e^	n.d.	1.34 ± 0.01 ^e–i^	0.07 ± 0.01 ^i–k^	0.28 ± 0.01 ^g,h^	5.12 ± 0.26 ^j^	0.01 ± 0.00 ^b^	n.d.
Summit	0.22 ± 0.01 ^b^	0.47 ± 0.01 ^b^	1.73 ± 0.09 ^c^	0.20 ± 0.01 ^c^	0.38 ± 0.04 ^d–f^	11.89 ± 0.03 ^c,d^	n.d.	0.06 ± 0.00 ^c^
Heritage	0.13 ± 0.01 ^g^	n.d.	2.37 ± 0.26 ^a^	0.09 ± 0.02 ^f–h^	0.58 ± 0.05 ^b^	13.85 ± 2.38 ^a^	0.02 ± 0.00 ^a^	n.d.
Bulgaskc	0.19 ± 0.01 ^c,d^	0.19 ± 0.00 ^h^	1.59 ± 0.06 ^c–e^	0.16 ± 0.00 ^d^	0.76 ± 0.07 ^a^	2.95 ± 0.19 ^l^	0.01 ± 0.00 ^b^	0.02 ± 0.00 ^d^
Samodiva	0.28 ± 0.01 ^a^	0.30 ± 0.01 ^e^	2.02 ± 0.07 ^b^	0.17 ± 0.01 ^d^	0.82 ± 0.02 ^a^	6.28 ± 0.12 ^i^	0.01 ± 0.00 ^b^	n.d.
Schopska	0.16 ± 0.01 ^e,f^	0.20 ± 0.01 ^h^	2.06 ± 0.04 ^b^	0.07 ± 0.00 ^h–k^	0.28 ± 0.03 ^g,h^	12.41 ± 0.31 ^b,c^	0.01 ± 0.00 ^b^	n.d.
Beijing10	0.22 ± 0.03 ^b^	0.61 ± 0.01 ^a^	1.67 ± 0.40 ^c,d^	0.10 ± 0.02 ^f^	n.d.	13.47 ± 0.49 ^a,b^	0.01 ± 0.00 ^b^	n.d.
Beijing19	0.19 ± 0.00 ^c,d^	0.39 ± 0.00 ^c^	1.39 ± 0.02 ^d–h^	0.07 ± 0.01 ^i–k^	0.05 ± 0.01 ^j^	9.15 ± 0.91 ^f,g^	0.01 ± 0.00 ^b^	n.d.
Beijing21	0.08 ± 0.01 ^h^	n.d.	1.11 ± 0.36 ^h,i^	0.07 ± 0.00 ^h–k^	0.43 ± 0.01 ^c,d^	8.08 ± 0.02 ^g,h^	0.01 ± 0.00 ^b^	n.d.
Beijing32	0.06 ± 0.00 ^i^	n.d.	1.23 ± 0.04 ^f–i^	0.07 ± 0.01 ^i–k^	0.42 ± 0.02 ^c–e^	11.37 ± 0.05 ^c,d^	n.d.	0.22 ± 0.01 ^b^
Fertod zamatos	0.16 ± 0.01 ^e^	0.25 ± 0.01 ^g^	1.53 ± 0.06 ^c–f^	0.26 ± 0.01 ^a^	n.d.	11.67 ± 0.08 ^c,d^	0.01 ± 0.00 ^b^	n.d.
Willamette	0.17 ± 0.01 ^d,e^	0.09 ± 0.01 ^i^	1.22 ± 0.09 ^g–i^	0.08 ± 0.00 ^f–i^	0.06 ± 0.01 ^j^	11.75 ± 0.27 ^c,d^	0.02 ± 0.00 ^a^	n.d.

Values are given as the mean ± standard deviation (*n* = 3), and the different letters within each column were significantly different (*p* < 0.05). n.d.: Not detected.

## Data Availability

Data is contained within the article.

## Supplementary Materials

The following supporting information can be downloaded at: https://www.mdpi.com/article/10.3390/foods11203232/s1, Table S1: Eigenvalue, variance contribution rate and cumulative variance contribution rate of the 7 principal components; Table S2: Principal component values and comprehensive evaluation indexes of 24 red raspberry varieties.
