# Supplementary material for: Comprehensive Evaluation of 24 Red Raspberry Varieties in Northeast China Based on Nutrition and Taste
Source: Foods. 2022 Oct 16;11(20):3232. doi: 10.3390/foods11203232 (PMC9601403; doi:10.3390/foods11203232)
Supplement: Supplementary file 1 [file foods-11-03232-s001.zip › foods-1950775-supplementary.pdf]

Table S1. Eigenvalue, variance contribution rate and cumulative variance contribution rate of the 7 principal components.

Indexes	PC1	PC2	PC3	PC4	PC5	PC6	PC7
l-Rhamnose monohydrate	−0.016	−0.605	−0.309	−0.070	−0.314	0.091	0.315
Fructose	−0.360	−0.722	0.188	0.043	0.063	0.008	0.226
Glucose	−0.024	−0.860	0.388	−0.085	−0.066	0.033	−0.200
Sucrose	0.773	0.223	−0.393	−0.246	0.022	0.025	0.070
Maltose	0.417	−0.053	−0.473	−0.593	0.130	0.107	−0.011
d-Trehalose anhydrous	−0.210	−0.167	−0.204	−0.238	0.389	−0.174	−0.727
Oxalic acid	−0.199	−0.190	−0.416	−0.045	−0.507	−0.565	−0.180
Tartaric acid	−0.134	−0.015	−0.046	0.320	−0.648	−0.474	−0.300
Malic acid	−0.399	−0.382	−0.735	−0.229	0.015	0.021	−0.097
α-Ketoglutaric acid	0.240	0.058	−0.441	−0.193	−0.543	0.336	−0.309
Lactic acid	0.378	−0.056	−0.254	−0.637	−0.292	0.336	−0.134
Citric acid	−0.898	−0.127	−0.248	0.149	0.096	0.195	0.026
Fumaric acid	−0.211	−0.389	−0.144	0.027	0.592	−0.392	0.021
Succinic acid	−0.063	0.346	0.605	−0.341	−0.027	0.017	−0.082
Total sugar	0.360	−0.666	0.051	−0.409	0.034	0.066	0.006
Reducing sugar	−0.230	−0.643	0.517	−0.264	−0.282	−0.047	−0.027
TAC	−0.927	−0.113	−0.233	0.059	−0.026	0.141	−0.029
SAR	0.906	0.064	−0.093	−0.107	0.107	−0.326	0.017
SSC	0.271	−0.551	−0.535	−0.049	0.131	−0.197	0.126
pH	0.947	0.031	0.101	−0.155	0.057	−0.171	−0.039
Moisture	−0.389	0.577	0.091	−0.344	−0.201	−0.142	−0.148
Length	−0.598	0.497	0.120	−0.500	−0.006	−0.219	0.008
Diameter	−0.851	0.056	−0.231	−0.287	0.137	−0.013	0.004
Weight	−0.696	0.342	−0.154	−0.450	−0.036	−0.039	0.147
Ascorbic acid	0.085	0.003	−0.398	0.717	0.174	0.088	−0.197
TPC	−0.024	−0.860	0.388	−0.085	−0.066	0.033	−0.200
Eigenvalue	6.776	4.802	3.162	2.675	1.902	1.294	1.118
Variance contribution rate (%)	26.06	18.47	12.16	10.287	7.316	4.975	4.299
Cumulative variance contribution rate (%)	26.06	44.53	56.69	66.978	74.293	79.268	83.567

Table S2. Principal component values and comprehensive evaluation indexes of 24 red raspberry varieties.

Varieties	PCA							Evaluation index value		Score ranking	
	PC1	PC2	PC3	PC4	PC5	PC6	PC7	Y_c	Y_f	Y_c	Y_f
European red	1.850	1.623	−0.301	1.205	1.279	−1.649	0.048	0.883	0.482	1	2
Tulameen	0.510	−0.798	0.889	0.422	−0.313	0.506	−0.673	0.110	0.133	9	6
Boyne	−0.252	0.468	−0.149	0.726	0.363	0.405	1.723	0.198	−0.066	7	10
DNS1	−0.334	−0.421	0.523	−0.105	−0.573	−0.167	−0.950	−0.203	−0.087	14	12
DNS2	−0.334	−2.220	−0.218	0.172	0.707	0.200	1.825	−0.366	−0.087	23	12
DNS4	0.142	0.692	3.224	−0.966	0.139	−1.049	−0.926	0.376	0.037	3	8
DNS5	−0.990	0.768	−0.646	−0.690	−1.778	0.153	1.095	−0.341	−0.258	22	21
DNS9	2.230	1.209	−0.366	0.742	−1.038	1.498	−0.370	0.819	0.581	2	1
Ruby	−0.695	0.695	0.632	−0.102	−0.073	−1.039	0.363	−0.028	−0.181	12	18
Royalty	−0.561	−0.666	0.882	−0.231	−0.135	−0.100	−0.412	−0.218	−0.146	16	15
Rerille	−0.922	0.984	−0.565	−0.383	−0.563	−1.667	0.139	−0.285	−0.240	18	20
Nootka	−0.789	0.794	−0.192	−0.882	−0.372	0.540	0.841	−0.137	−0.206	13	19
Canby	1.612	−0.479	−0.246	−0.775	1.171	−0.178	0.646	0.327	0.420	4	4
Summit	−0.351	−0.344	−0.573	0.490	−1.720	0.575	−0.749	−0.304	−0.091	20	13
Heritage	−1.107	0.056	−1.700	−1.887	2.237	0.149	−2.273	−0.606	−0.288	24	22
Bulgaskc	1.747	−0.625	−0.643	−1.295	−0.408	0.013	0.214	0.108	0.455	10	3
Samodiva	1.132	−0.927	−1.394	−1.315	−1.160	−1.081	−0.239	−0.330	0.295	21	5
Schopska	−0.678	−1.167	−0.251	0.629	0.590	0.896	−0.018	−0.271	−0.177	17	17
Beijing10	−1.127	0.171	−0.337	1.335	−0.862	−0.931	−0.488	−0.296	−0.294	19	23
Beijing19	0.038	−1.626	0.665	0.647	−0.367	−1.068	0.192	−0.215	0.010	15	9
Beijing21	0.281	−0.756	1.346	0.291	0.706	1.467	−0.308	0.239	0.073	6	7
Beijing32	−0.467	1.644	0.637	−1.273	0.280	2.166	0.836	0.293	−0.122	5	14
Fertod zamatos	−0.328	0.382	−0.817	2.111	0.191	0.884	−1.786	0.084	−0.085	11	11
Willamette	−0.605	0.541	−0.399	1.133	1.698	−0.523	1.269	0.163	−0.158	8	16

Y_c: The comprehensive quality score. Y_f: The comprehensive evaluation score of fresh utilization.
